# Einfluss von Konservierungsmitteln in Topika auf die kutane Mikrobiota

**DOI:** 10.1007/s00105-023-05112-x

**Published:** 2023-02-02

**Authors:** Kathrin Richter, Johannes Wohlrab

**Affiliations:** 1 Universitätsklinik und Poliklinik für Dermatologie und Venerologie, Martin-Luther-Universität Halle-Wittenberg, Ernst-Grube-Straße 40, 06114 Halle (Saale), Deutschland; 2 An-Institut für angewandte Dermatopharmazie, Martin-Luther-Universität Halle-Wittenberg, Halle (Saale), Deutschland

**Keywords:** Kutanes Mikrobiom, Mikrobiota, Konservierungsmittel, Vehikelsystem, Vehikelmetamorphose, Cutaneous microbiome, Microbiota, Preservatives, pharmaceutical, Vehicle system, Metamorphosis of vehicle

## Abstract

Konservierungsmittel dienen der Haltbarmachung topischer Präparate und schützen dadurch den Anwender vor dem Einfluss pathogener Mikroben. Mit der Applikation des Topikums durchläuft die Matrix eine Metamorphose, in deren Folge es, durch anteilige Volatilisierung der hydrophilen Phase, zur Anreicherung des Konservierungsmittels auf der Hautoberfläche kommen kann. Es wird angenommen, dass dies zu antiseptischen Effekten und der Beeinflussung der Diversität der kutanen Mikrobiota führt. Wegen der Komplexität der Regulation des kutanen Mikrobioms und der damit verbundenen Einflussfaktoren resultiert ein hoher Grad der Individualisierung, sodass Untersuchungen zum Einfluss von definierten Interventionen methodisch schwierig sind. In der vorliegenden „Proof-of-concept“-Studie wurden mögliche antiseptische Effekte von Konservierungsmitteln in einer Kombination von In-vitro- und In-vivo-Methoden mittels mikrobiologischer Kulturversuche untersucht. Darüber hinaus dienten die Untersuchungen der Erarbeitung eines klinischen Studiendesigns für weiterführende Fragestellungen und erweitertem Methodenspektrum.

Die Ergebnisse stützen die Hypothese einer antiseptischen Wirkung der getesteten Konservierungsmittel (Methyl-4-hydroxybenzoat und Propyl-4-hydroxybenzoat, Sorbinsäure/Kaliumsorbat und Propylenglykol) auf prominente Referenzbakterien und konnten auch im klinischen Setting beobachtet werden.

## Abstract (en)

Preservatives are used to stabilize topical preparations and protect the user from the influence of pathogenic microbes. After the application of a topical preparation, the matrix undergoes a metamorphosis, and by proportional evaporation of the hydrophilic phase the preservative may accumulate on the skin surface. This is believed to lead to antiseptic effects and may influence the diversity of the cutaneous microbiota. The regulation of the cutaneous microbiome and the associated influencing factors is a complex system that results in highly individualized conditions. Therefore, investigations on the influence of defined interventions are methodologically difficult. In the present proof-of-concept study, potential antiseptic effects of preservatives were investigated in a combination of in vitro and in vivo methods using microbiological culture tests. In addition, the investigations served to develop a clinical study design to answer further questions and use of an extended range of methods. The results support the hypothesis of an antiseptic effect of the tested preservatives (methyl-4-hydroxybenzoate and propyl-4-hydroxybenzoate, potassium sorbate and propylene glycol) on prominent reference bacteria, which could also be observed in clinical settings.

## Einführung

In den letzten Jahren ist die Bedeutung der kutanen Mikrobiota (Gesamtheit aller auf der Haut lebenden Mikroorganismen) für die Homöostase des Hautorgans immer deutlicher geworden [1, 2]. Sie wird heute als ein diverses Ökosystem verstanden, welches hauptsächlich aus grampositiven Bakterien besteht und die menschliche Haut je nach Region und Individuum in unterschiedlicher Dichte und Zusammensetzung besiedelt [1, 3]. So wird die kutane Mikrobiota als funktionaler und aktiver Teil der epidermalen Barriere aufgefasst, da sie sowohl dem Wachstum potenziell pathogener Bakterien entgegenwirkt als auch mit fremden Mikroorganismen kommuniziert und darüber hinaus mit dem Immunsystem des Wirts interagiert [4, 5]. Dieses Ökosystem wird von genetischen Komponenten geprägt, maternal erworben und im Laufe des Lebens adaptiert [6, 7]. Die funktionelle Verzahnung dieser Symbiose wird besonders dann deutlich, wenn es durch pathogenetische Veränderungen zu einer relevanten Dysbiose und daraus folgend zu einer phänotypischen Erkrankung kommt.

Das Bewusstsein für diese Zusammenhänge ist sowohl auf die Fortschritte in der molekularen Analytik zur Identifizierung des kutanen Mikrobioms als auch auf ein erweitertes immunologisches Verständnis zur funktionellen Bedeutung der Interaktionsmuster von Mikrobiota und Wirt zurückzuführen [8, 9]. Insbesondere durch spezielle molekularbiologische Technologien wie die Metagenomsequenzierung oder das Next Generation Sequencing (NGS) werden eine genaue taxonomische Zuordnung identifizierter Arten sowie eine Charakterisierung der Diversität des Mikrobioms ermöglicht [10]. Im Rahmen der NGS wird eine Analyse der bakteriellen DNA durchgeführt, die Bezug auf hochkonservierte und hochvariable Regionen des *16S-rRNA*-Gens als Teil des bakteriellen Ribosoms nimmt [11]. Das zur Sequenzierung verwendete PCR-Produkt wird durch spezifische Primer erzeugt, die an die hochkonservierten Regionen in unmittelbarer Nachbarschaft der variablen Regionen des *16S-rRNA*-Gens der Bakterien binden. Anschließend wird das generierte Amplifikat sequenziert, und die dabei ermittelten Sequenzvarianten werden durch bioinformatische Analysen mit *16S-rRNA*-Gen-Sequenzen bekannter Bakterien aus Datenbanken verglichen und taxonomisch klassifiziert [12]. Probleme dieser Analysen liegen in der Auswahl der variablen Regionen sowie im Umstand, dass die Methodik und das Material der Probennahme sehr fehleranfällig sind, begründet [13, 14]. Deshalb wird der parallele Einsatz eines zweiten Nachweisverfahrens, wie z. B. der Matrix-Assisted Laser Ionization Time-of-Flight Mass Spectroscopy (MALDI-TOF MS), propagiert [15–17]. Diese Herangehensweise steigert allerdings den zeitlichen und finanziellen Aufwand von Studien erheblich, ist aber dem parallelen Einsatz konventioneller mikrobiologischer Kulturtechniken sehr wahrscheinlich überlegen [18]. Das Mikrobiom ist ein extrem komplexes und intra- sowie interindividuell variables System, welches zudem von vielen intrinsischen und extrinsischen Faktoren beeinflusst werden kann [19, 20]. Mittels *16S rRNA*-Sequenzierung lässt sich das genetische Material der Bakterien analysieren, jedoch können mit dieser Methode keine Aussagen über den Vitalitätszustand der Mikroben getroffen werden, was Untersuchungen zu antiseptischen Effekten von Konservierungsmitteln methodisch schwierig macht [21, 22].

Es ist zudem unklar, wie lange nach einer Anwendung mikrobizider Präparate auf der Hautoberfläche genetisches Material der avitalen Mikroben persistiert und damit molekularbiologisch erfasst werden kann [23, 24]. Folglich ist die Analyse von antiseptischen Effekten nach Interventionen mittels *16S-rRNA*-Sequenzierung nur eingeschränkt möglich. Im Gegensatz dazu bieten klassische Kulturverfahren zwar die Möglichkeit, einzelne Bakterienarten zu analysieren, lassen aber keine Aussagen zur Diversität der Mikrobiota zu und sind von verwendeten Kulturmedien, Kulturbedingungen und der Wachstumsdynamik der jeweiligen Art (Zeit-Überleben-Kurve) abhängig. Das Wachstumsverhalten von Bakterien wird nach dem Beimpfen der Kulturplatten durch die Adaptation an die Umgebungsbedingungen durch eine Latenzphase („lag phase“) geprägt [25]. Anschließend erfolgt ein exponentielles Wachstum („log phase“), welches nach Übergang in eine stationäre Phase schließlich in eine regressive Phase übergeht. Die genaue Dauer der einzelnen Phasen ist v. a. von der Ausgangskeimzahl und den Wachstumseigenschaften der Bakterienart, aber auch von den Umgebungsbedingungen abhängig. Bei der Planung von Kulturversuchen mit Referenzbakterien müssen diese Faktoren Berücksichtigung finden, um relevante Ergebnisse ableiten zu können. Der Einsatz von Konservierungsmitteln ist in der Lag phase besonders relevant, da die Log phase verhindert und ein Übergang in die stationäre (mikrobistatisch) bzw. regressive (mikrobizid) Phase induziert wird [26]. Daraus lässt sich die Sinnhaftigkeit des Einsatzes von Konservierungsmitteln in Topika ableiten.

### Konservierung von Topika

Konservierungsmittel werden als Hilfsstoffe in Topika eingesetzt, um die jeweilige Präparation mikrobiologisch haltbar zu machen und damit zu einer möglichst langen Lager- und „In-use“-Stabilität beizutragen, darüber hinaus aber auch, um den Anwender des Topikums vor unerwünschten Einflüssen pathogener und apathogener Mikroorganismen zu schützen [27, 28]. Der Einsatz von Konservierungsmitteln wird bezüglich der Auswahl geeigneter chemischer Stoffe oder Stoffgemische mit mikrobistatischer oder mikrobizider Aktivität sowie deren Anwendungskonzentration regulatorisch definiert [29]. Sie besitzen vordergründig eine mikrobistatische Wirkung, die abhängig von der Konzentration und der spezifischen Reaktionsweise der zugehörigen Substanzgruppe ist [30]. Physikochemisch besitzen Konservierungsmittel typischerweise einen amphiphilen Charakter, sind gut wasserlöslich und können über den lipophilen Anteil an Membranen anhaften sowie diese passiv durchdringen. An der Zellmembran von Mikroorganismen kommt es zur konzentrationsabhängigen Permeationsstörung und im Falle einer mikrobiziden Konzentration zum Zelltod bzw. sogar zur Auflösung der kolloidphysikalischen Ordnung der Zelle und damit zu deren Autolyse. Die als Konservierungsmittel zugelassenen Substanzen unterliegen einem definierten Konzentrationsbereich und stammen aus den Substanzklassen der quartären Stickstoffverbindungen, Carbonsäuren, Alkohole, Phenole, Organoquecksilberverbindungen sowie sonstiger Stoffe [26, 31]. Konservierungsmittel wirken meistens im undissoziierten Zustand, sodass der pH-Wert der hydrophilen Phase, in Abhängigkeit von der pH-Toleranz relevanter Bakterienarten, direkten Einfluss auf die Wirkeffizienz nimmt. Die individuelle minimale Hemmkonzentration (MHK) entspricht weitestgehend der mikrobistatischen Konzentration [32]. Konservierungsmittel werden durch Kontamination verbraucht, sodass die MHK bei entsprechend hoher mikrobieller Last unterschritten werden kann und die mikrobistatische Wirkung ausbleibt. Die Abtötungsgeschwindigkeitskonstante bei mikrobizider Wirkung eines Konservierungsmittels ist stark von der Zahl und der Art der lebensfähigen Mikroorganismen und der Einwirkzeit des Konservierungsmittels abhängig. Aus regulatorischer Sicht wird die Einsatzkonzentration eines Konservierungsmittels nicht nur aus mikrobiologischer Perspektive bewertet [31]. So werden ebenso die toxikologische und allergologische Unbedenklichkeit und die chemische Inaktivität der zulässigen Substanzen innerhalb topischer Matrices beurteilt [33]. Der zugelassene Konzentrationsbereich wird dabei v. a. an der MHK innerhalb der Formulierungen im Primärpackmittel ausgerichtet (mikrobiologische Stabilität; [26, 29]). Da Bakterien Wasser zum Wachstum benötigen, ist bei keimarm hergestellten galenischen Systemen v. a. dann eine Konservierung erforderlich, wenn sie eine äußere kontinuierliche hydrophile Phase aufweisen.

### Von der Konservierung zur Antiseptik

Bisher wenig untersucht sind die antiseptischen Effekte von Konservierungsmitteln nach epikutaner Applikation auf die kutane Mikrobiota. Aus pharmakokinetischer Sicht ergeben sich aus den Kenntnissen der Konversion der Applikationsmatrix in die Segregationsmatrix des Vehikelsystems wesentliche Anhaltspunkte dafür, dass sich das Konzentrationsniveau der Konservierungsmittel auf der Haut nach der Applikation erheblich erhöhen kann. Während der strukturellen Umwandlung der Applikationsmatrix (Metamorphose) und der sich dabei vollziehenden Verdunstung volatiler Bestandteile der Formulierungsmatrix, zu denen auch Wasser gerechnet werden muss, kommt es zum Entzug des Lösungsmittels der Konservierungsmittel und damit zu deren Konzentrierung auf der Hautoberfläche [34]. Daraus ergibt sich zwangsläufig, dass die Konservierungsmittel auch Einfluss auf die kutane Mikrobiota nehmen. Zudem sind bei der überwiegenden Zahl der Topika, insbesondere bei Arzneimitteln, weder der pH-Wert der Wasserphase noch die Pufferkapazität an den pH-Gradienten des Stratum corneum und damit an das physiologische Milieu der kutanen Mikrobiota angepasst [35]. Während der Einfluss des pH-Wertes und der Pufferkapazität in der Literatur durchaus als relevant für die Mikrobiota diskutiert wird, werden die Konzentrierung der Konservierungsmittel im Zuge der Matrixkonversion und deren Folgen für die Mikrobiota bisher selten adressiert [36]. Es finden sich lediglich Daten zu einzelnen kosmetischen Produkten (überwiegend „Wash-off“-Präparaten), die im Studiensetting die Remanenzeigenschaften der eingesetzten Konservierungsmittel vernachlässigen und ausschließlich *16S-rRNA*-Analysen betrachten [36]. Die bisher vorliegenden Daten lassen deshalb keine objektive Bewertung über die Einflussnahme von Konservierungsmitteln auf die kutane Mikrobiota zu, besonderes bei „Leave-on“-Präparationen. Dies liegt auch an den sehr komplexen Zusammenhängen und Einflussfaktoren, die sich auf mikrobiologischer, pharmakokinetischer, pharmakodynamischer und galenischer Ebene darstellen. Deshalb bedarf es mehrerer methodischer Ansätze, die die verschiedenen Konservierungsstrategien untersuchen, um insbesondere die praktische Relevanz antiseptischer Effekte von Konservierungsmitteln besser bewerten zu können. Die vorliegenden Untersuchungen sollen deshalb anhand von Kulturversuchen an Referenzbakterien mit dermatologischer Relevanz zeigen, dass für Topika gängige Konservierungsmittel in ansonsten mikrobiologisch inerten Matrizes grundsätzlich einen direkten Einfluss auf die Mikrobiota in Form antiseptischer Effekte ausüben können. Die daraus resultierenden Erkenntnisse sollen darüber hinaus der Erarbeitung klinischer Studiendesigns dienen, um eine weiterführende Untersuchung des Einflusses von Konservierungsmitteln auf die kutane Mikrobiota zu ermöglichen.

## Material und Methoden

### Auswahl von Referenzbakterien

Die 3 kutan bedeutsamen eu- oder dysbiotischen Bakterienarten: *Staphylococcus aureus* (DSM no. 6148, WS 1759; [37, 38]), *Pseudomonas aeruginosa* (DSM no. 1128, ATCC 9027; [39, 40]) und *Corynebacterium xerosis* (DSM no. 20743, ATCC 373; [41–46]) wurden aufgrund ihres Kulturverhaltens für die geplanten Untersuchungen ausgewählt. Die Kultur der Referenzbakterien erfolgte für *Staphylococcus aureus* auf Chapman-Agar (Oxoid® Tergitol-7-Agar; Fa. Thermo Fisher Scientific GmbH, Schwerte, Deutschland), für *Pseudomonas aeruginosa* auf Cetrimid-CN-Agar (Pseudomonas CN Selectiv-Agar; Fa. Thermo Fisher Scientific GmbH, Schwerte, Deutschland) und für *Corynebacterium xerosis* in Casein-Soja-Pepton-Agar (Oxoid® TSA; Fa. Thermo Fisher Scientific GmbH, Schwerte, Deutschland). Die Referenzbakterien wurden in Suspension auf den McFarland-Standard 0,5 (McFarland-Trübungsstandards und Wickerham-Karte; Fa. Bioanalytic GmbH, Umkirch, Deutschland) eingestellt und für die weitere Verwendung entsprechend verdünnt [47].

### Auswahl von Konservierungsmitteln

Das *Europäisches Arzneibuch* sieht definierte Substanzen zur Konservierung halbfester Zubereitungen vor. Dessen Einsatz richtet sich nach den Akzeptanzkriterien für die mikrobiologische Qualität nichtsteriler Darreichungsformen [29]. Für die vorliegenden Untersuchungen wurden Konservierungsmittel verwendet, die als gängige Konservierungsstrategien in Topika eingesetzt werden. Ausgewählt wurden ein Phenolgemisch aus Methyl-4-hydroxybenzoat (MHB; Fa. Caesar & Loretz GmbH, Hilden, Deutschland) und Propyl-4-hydroxybenzoat (PHB; Fa. Caesar & Loretz GmbH, Hilden, Deutschland; 0,05 %; 0,075 %; 0,1 % w/w), Sorbinsäure und Kaliumsorbat als Salz der Sorbinsäure (0,05 %; 0,1 %; 0,2 % w/w) sowie aus der Gruppe der Alkohole Propylenglykol (Fa. Caesar & Loretz GmbH, Hilden, Deutschland; 10 %; 20 %; 30 % v/v). Die untersuchten Konzentrationen wurden nach dem für die Anwendung zugelassenen Konzentrationsbereichen festgelegt.

### Auswahl eines Vehikelsystems

Als Vehikelsystem für die Konservierungsmittel wurde als einheitliche, mikrobiologisch inerte Matrix für die In-vitro-Untersuchungen ein 3 %iges Hydroxyethylcellulose(HEC)-Gel (Fa. Caesar & Loretz GmbH, Hilden, Deutschland) mit Phosphatpuffer (Kaliumdihydrogenphosphat; Fa. Merck, Darmstadt, Deutschland, und Dinatriumhydrogenphosphat; Fa. Carl-Roth GmbH & Co. KG, Karlsruhe, Deutschland) verwendet, welches auf einen pH-Wert von 5,5 eingestellt wurde und damit dem pH-Optimum aller verwendeten Konservierungsmittel entspricht. Diese wurden in den entsprechenden Konzentrationen in das unkonservierte Vehikelsystem eingearbeitet und im Anschluss autoklaviert.

### Experimentelles Design der In-vitro-Untersuchungen

Zur Untersuchung des Wachstums der ausgewählten Referenzbakterienarten unter Einfluss der Konservierungsmittel wurde eine experimentelle In-vitro-Strategie erarbeitet.

Entsprechend der gängigen Kultivierungsmethodik wurden 3 Interventionsschritte definiert: Medium, Bakterien und Kultur. Um den Einfluss der Konservierungsmittel auf den Kultivierungsvorgang zu untersuchen, wurde das jeweilige Konservierungsmittel in einer Konzentrationsreihe 1. dem Medium (Mediumkonservierungstest, MKT), 2. einer definierten Keimzahl von Bakterien (Vehikelkonservierungstest, VKT) und 3. der angewachsenen Kultur (Plattendiffusionstest, PDT) hinzugegeben und anschließend dessen antimikrobiellen Effekte quantifiziert. Als Negativkontrolle wurden das unkonservierte, inerte Vehikelsystem, physiologische Natriumchloridlösung (Fa. B. Braun Melsungen AG, Melsungen, Deutschland) und Aqua bidestillata verwendet. Als Positivkontrollen diente eine wässrige 2 %ige Chlorhexidinlösung (Fa. Sigma-Aldrich, Darmstadt, Deutschland).

### Definition von Parametern

Im MKT und VKT wurden die koloniebildenden Einheiten (KbE) sowie der prozentuale Anteil der kolonisierten Fläche an der Gesamtfläche des Festmediums als Maß für das Bakterienwachstum bestimmt. Im PDT wurde hingegen der unbesiedelte Hof um die Testplättchen als Maß der antimikrobiellen Wirksamkeit ermittelt. Alle Parameter wurden jeweils nach 24 und 48 h erfasst und dokumentiert.

### Mediumkonservierungstest

**Figure 1:**
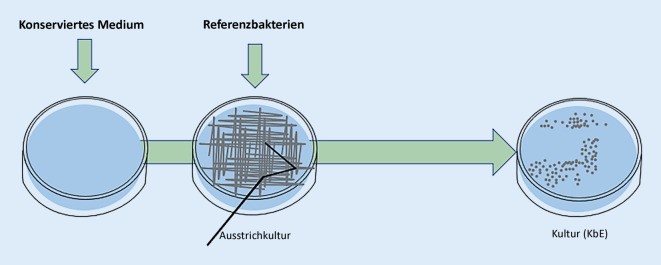

Im MKT wurde der Einfluss des Konservierungsmittels auf das Anwachsen der Kultur auf einem dafür geeigneten Medium untersucht. Dazu wurden die jeweiligen Konservierungsmittel in Luria-Bertani-Medium (LB-Agar, Trypton 10 g/l, Hefeextrakt 5 g/l, Natriumchlorid 10 g/l, Agar-Agar 15 g/l; Fa. Carl-Roth GmbH & Co. KG, Karlsruhe, Deutschland), auf einen pH-Wert von 5,5 eingestellt und nach Autoklavierung als Plattenmedium ausgegossen. Die Referenzbakterienarten wurden in einer sterilen physiologischen Kochsalzlösung suspendiert (McFarland-Standard 0,5) und zu je 100 µl Bakteriensuspension auf die präparierte Agarplatte in einer Verdünnung von 1:100 (*Staphylococcus aureus*), von 1:1000 (*Pseudomonas aeruginosa*) und von 1:10 (*Corynebacterium xerosis*) aufgebracht und mit einem sterilisierten Drigalski-Spatel gleichmäßig verteilt (Abb. 1).

### Vehikelkonservierungstest

**Figure 2:**
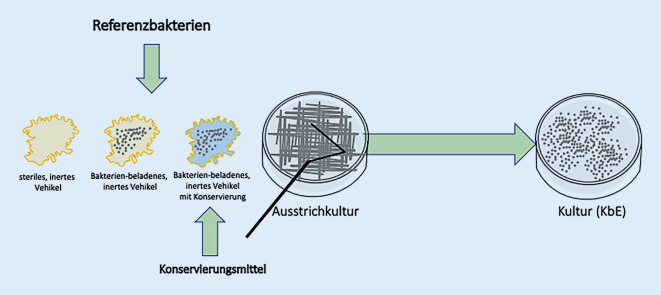

Der VKT diente der Bestimmung der bakteriellen Wachstumshemmung in einem konservierten Vehikelsystem. Dazu wurden 200 µl des konservierten Vehikels auf das jeweilige Kulturmedium aufgebracht und mit einem sterilen Drigalski-Spatel gleichmäßig verteilt. Anschließend wurde 100 µl Bakteriensuspension (McFarland-Standard 0,5) auf die mit dem konservierten Vehikel beschichtete Agarplatte in einer Verdünnung von 1:100 (*Staphylococcus aureus*), von 1:1000 (*Pseudomonas aeruginosa*) und von 1:10 (*Corynebacterium xerosis*) ausgegossen, durch Schwenken der Platte gleichmäßig verteilt und mittels Drigalski-Spatel mit diesem vermischt sowie anschließend kultiviert (Abb. 2).

### Plattendiffusionstest

**Figure 3:**
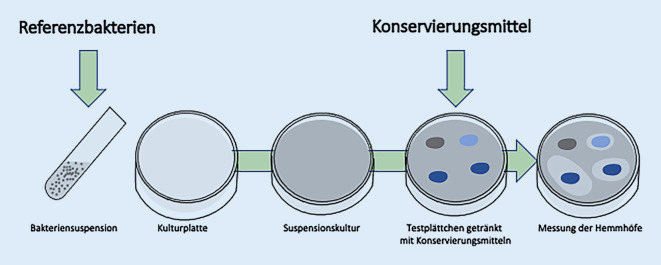

Der PDT, der auch als Hemmhoftest bekannt ist, untersucht den Einfluss von Konservierungsmitteln auf eine floride Bakterienkultur. Dazu wurden die jeweiligen Bakterienarten in Casein-Soja-Pepton-Medium (Trypton-Soja-Bouillon; Fa. Merck-Millipore, Darmstadt, Deutschland) vorkultiviert. Anschließend wurde eine volle Impföse aus einer der Bakterienkulturen in 5 ml einer physiologischen Natriumchloridlösung eingebracht und die Bakteriensuspension auf Casein-Soja-Pepton-Agar (Oxoid® TSA; Fa. Thermo Fisher Scientific GmbH, Schwerte, Deutschland) ausgegossen, durch Schwenken der Platte gleichmäßig verteilt, der Überstand abgegossen und die Bakteriensuspension für ca. 3 min antrocknen lassen. Dann wurden wässrige Lösungen der Konservierungsmittel in Konzentrationsreihen sowie die Kontrollen mittels darin getränkter (5 µl) steriler Zellulosepapierplättchen (Cytiva Whatman® Filterpapier Gütegrad 1, Durchmesser 5 mm, Dicke 180 µm, Porengröße 11 µm, Grundgewicht 87 g/m^2^; Sigma-Aldrich Inc., Saint Louis, USA) auf der Platte strukturiert platziert. Die dabei durch Diffusion der antimikrobiellen Testsubstanzen entstandenen Hemmhöfe wurden in ihrem Durchmesser, als Maß des antimikrobiellen Effektes, erfasst (Abb. 3).

### Design der In-vivo-Untersuchung („Proof-of-concept“-Studie)

Mit positivem Votum (2020-063) der Ethikkommission der Medizinischen Fakultät der Martin-Luther-Universität Halle-Wittenberg vom 30.07.2020 wurde die Proof-of-concept-Studie „K03/20-IIT – Prospektive, randomisierte, doppelt verblindete, monozentrische Studie zur Untersuchung des Einflusses von konservierten Topika auf das kutane Mikrobiom gesunder Probanden“ (Prüfplan fV03, 15.07.2020) durchgeführt. Das primäre Studienziel war es, Erkenntnisse zum Einfluss von Konservierungsmitteln in Topika auf die kutane Mikrobiota zu erlangen. Dazu wurden ohne Fallzahlschätzung 24 gesunde Proband:innen zwischen 18 und 40 Jahren eingeschlossen, die seit 4 Wochen keine systemische oder topische Medikation erhalten hatten, bei denen keine Unverträglichkeitsreaktionen gegenüber Inhaltstoffen von Topika bekannt waren und die bereit waren, Lifestyle-Restriktionen im Vorfeld der Studie umzusetzen. Hierzu zählten ein mindestens einwöchige Karenz hinsichtlich Kosmetika oder Pflegeprodukten, eine mindestens 3‑tägige Karenz hinsichtlich Seife und Shampoo sowie eine mindestens 24-stündige Karenz hinsichtlich Körper-Wasser-Kontakt. In der Studie diente ein 3%iges Hydrogel auf Xanthanbasis als Vehikelsystem, welches unkonserviert (Vehikelkontrolle) oder mit MHB (0,075 %) und PHB (0,025 %) (Konservierung 1), Sorbinsäure/Kaliumsorbat (0,2 %) (Konservierung 2) und Propylenglykol (20 %) (Konservierung 3) konserviert untersucht wurde. Auf dem Rücken der Proband:innen wurden paravertebral auf gleicher Höhe jeweils links- und rechtsseitig 5 Felder (6 × 3 cm) markiert. Am Studientag wurden die 4 Testpräparationen und ein Leerkontrollfeld den Testfeldern entsprechend einem Randomisierungsplan (Research Randomizer V4.0; http://www.randomizer.org/) zugelost.

**Figure 4:**
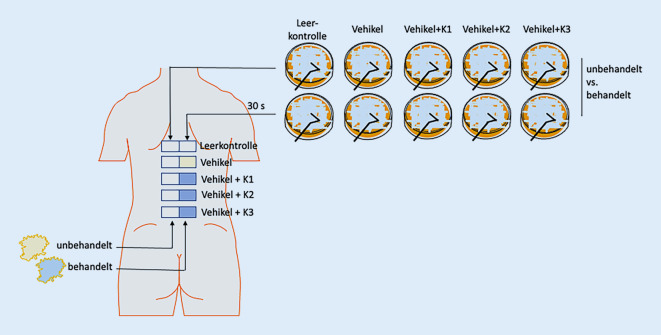

Eine Seite der Felder blieb als Leervergleich unbehandelt und abgestrichen. Auf der anderen Seite der Felder wurden 200 µl der jeweiligen niedrigviskösen Testpräparation durch Pipettierung appliziert, mit einem sterilen Plastikspatel (Fa. Carl-Roth GmbH & Co. KG, Karlsruhe, Deutschland) gleichmäßig verteilt und nach einer Applikationszeit von 30 s ein Abstrich entnommen. Dazu wurde ein, in physiologischer Natriumchloridlösung befeuchteter, steriler Abstrichtupfer (Viskosetupfer auf Polysterolträger; Fa. Sarstedt AG & Co. KG, Nümbrecht, Deutschland) verwendet, welcher 30 s lang fest und in Rollbewegungen über das markierte Areal gerieben wurde. Die Abstriche wurden anschließend zur Freisetzung der Mikroben aus dem Tupfer für 2 h bei 37 °C in einem Schüttelinkubator (200 rpm) in 500 µl Casein-Soja-Pepton-Medium (Trypton-Soja-Bouillon; Fa. Merck-Millipore, Darmstadt, Deutschland) vorinkubiert, auf eine vorgewärmte Casein-Soja-Pepton-Agarplatte (Oxoid® TSA; Fa. Thermo Fisher Scientific GmbH, Schwerte, Deutschland) ausgestrichen und bei 37 °C für 48 h bebrütet. Nach 24 und 48 h wurden die Kulturplatten unter der Laminarbox standardisiert fotografiert und über digitale Bildanalyse (ImageJ Java 1.8.0_172 Mac OS X, National Institutes of Health, Washington D.C., USA) die koloniebildenden Einheiten (KbE) und prozentual die kultivierte Fläche der Kulturplatte bestimmt (Abb. 4). Mittels Indexbildung wurden die jeweils intraindividuell korrespondierenden Werte ermittelt und interindividuell vergleichend dargestellt. Dabei wurde definiert, dass ein bakteristatischer Effekt als wahrscheinlich gelten kann, wenn die Kultur nach Probennahme von behandelten Arealen eine mindestens 20 % geringere Kultureffizienz bot als das intraindividuelle unbehandelte, korrespondierende Areal.

## Ergebnisse

### Mediumkonservierungstest

**Figure 5:**
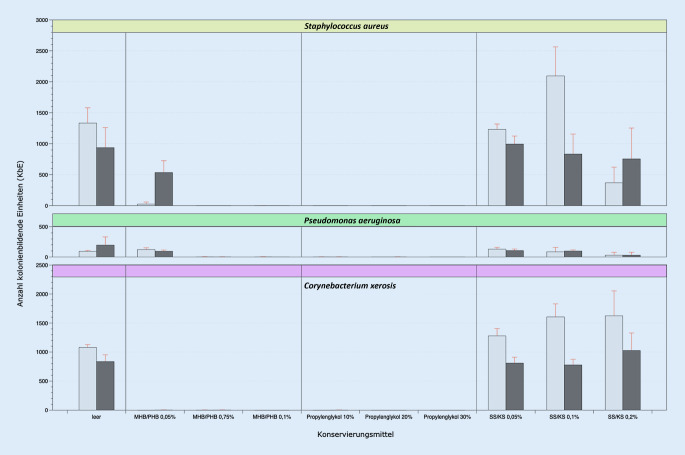

Im Rahmen des MKT wurde der Einfluss des Konservierungsmittels auf die Kultivierungsfähigkeit der Referenzbakterien getestet, wobei je nach Konservierungsmittel und eingesetzter Konzentration unterschiedliche Ergebnisse erzielt wurden. Die Konservierung mit Propylenglykol zeigte in allen getesteten Konzentrationen (10 %, 20 %, 30 % v/v) ein inhibierendes Wachstum auf die Bakterienkultur (Abb. 5). Ebenso ließ sich das Wachstum effektiv mittels der Parabenverbindung aus MHB/PHB in den eingesetzten Konzentrationen von 0,75 % und 0,1 % bei *S. aureus* und *P. aeruginosa* hemmen. Für das Referenzbakterium *C. xerosis* konnte bei dem Parabengemisch eine vollständige Hemmung bei allen Konzentrationsstufen (0,05 %, 0,75 %, 0,1 % w/w) ermittelt werden (Abb. 5). Kein bakteriostatischer Effekt konnte für das Sorbinsäure/Kaliumsorbat in allen Konzentrationen (0,05 %, 0,1 %, 0,2 % w/w) sowie getesteten Bakterienkulturen nachgewiesen werden (Abb. 5).

### Vehikelkonservierungstest

**Figure 6:**
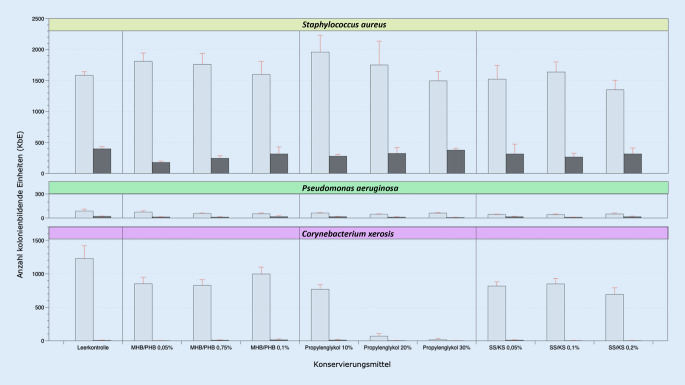

Im VKT konnte für alle 3 Konservierungsmittel konzentrationsunabhängig, bei allen Referenzbakterien übereinstimmend, kein bakteriostatischer Effekt dokumentiert werden (Abb. 6).

### Plattendiffusionstest

**Figure 7:**
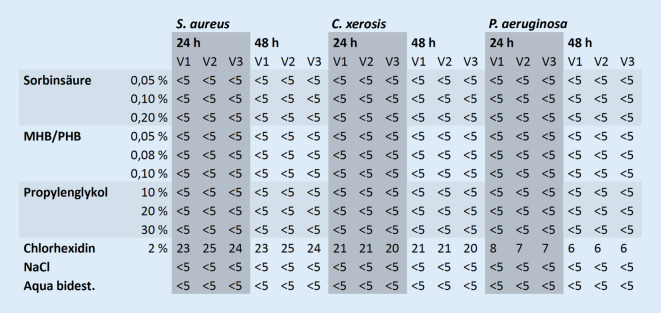

Die Durchführung des PDT zeigte unabhängig von den Einsatzkonzentrationen für alle Konservierungsmittel und Negativkontrollen keine Hinweise auf eine bakteristatische oder bakterizide Wirkung (Abb. 7).

### Proof-of-concept-Studie

**Figure 8:**
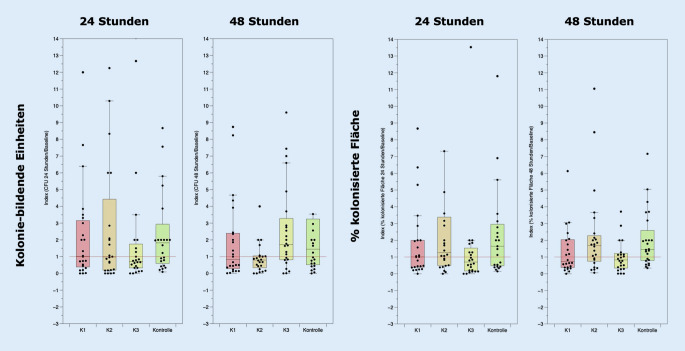

In die Studie wurden insgesamt 24 Proband:innen (m = 11, w = 13) im mittleren Alter von 29 Jahren (min = 21, max = 38) eingeschlossen, die der Teilnahme schriftlich zugestimmt hatten und den Ein- und Ausschlusskriterien des Studienprotokolls vollständig entsprachen. Die in der Kultur erfassten Parameter der Anzahl koloniebildender Einheiten (KbE) und die prozentuale kolonisierte Fläche zeigen in Abhängigkeit vom Konservierungsmittel eine mindestens 20 %ige Wachstumshemmung bei ca. 40–70 % der Proband:innen. Dabei konnte beim Parameter KbE für MHB/PHB (24/48 h) eine Wachstumshemmung bei 41,7/50,0 %, für Sorbinsäure/Kaliumsorbat bei 45,8/70,8 % und für Propylenglykol bei 58,3/37,5 % der Proben nachgewiesen werden (Abb. 8). Beim Parameter prozentual kolonisierte Fläche konnten vergleichbare Daten einer Wachstumshemmung für MHB/PHB (24/48 h) bei 58,3/54,2 %, für das Sorbinsäure/Kaliumsorbat bei 41,7/37,5 % sowie für Propylenglykol bei 54,2/54,2 % beobachtet werden (Abb. 8). Für die Indizes der Parameter nach 24 bzw. 48 h im Verhältnis zum Ausgangswert (Baseline) konnten keine statistischen Signifikanzen im Vergleich zur Kontrolle ermittelt werden (Einwegvarianzanalyse, *p* ≤ 0,05; Abb. 8). Die Daten zeigen eine sehr große Streuung, und die Effekte (Index < 1 ≙ Wachstumshemmung) sind für die untersuchten Konservierungsmittel nicht einheitlich, konzentrationsabhängig und regelmäßig nachweisbar.

## Diskussion

Zunächst bestätigen die vorliegenden Untersuchungsergebnisse die in der Einleitung dargestellten methodischen Schwierigkeiten der qualitativen und quantitativen Charakterisierung von antimikrobiellen Effekten von topischen Interventionen auf die kutane Mikrobiota [48]. Im Rahmen dieser Studie wurde festgestellt, dass die getesteten Konservierungsmittel in unterschiedlichem Ausmaß antimikrobielle Effekte auf die eingesetzten Referenzbakterien ausüben können. Anders als im klassischen Konservierungsansatz, bei dem keimarme oder sterile, flüssige oder halbfeste Zubereitungen mit den Konservierungsmitteln versetzt werden, um eine bakterielle Verunreinigung (sprich: das Anwachsen von Bakterien in der Zubereitung) zu verhindern, wurden in den vorliegenden Untersuchungen antiseptische Effekte bei einer vorgelegten bakteriellen Ausgangslast analysiert. Die vorliegenden Ergebnisse deuten darauf hin, dass in den In-vitro-Untersuchungen aufgrund der eliminationsabhängigen verminderten Bioverfügbarkeit der Konservierungsmittel und trotz der eingesetzten maximalen Konservierungsmittelkonzentration häufig kein oder nur ein geringer bakteristatischer Effekt nachgewiesen werden konnte. Dennoch wird in der Zusammenschau deutlich, dass in Abhängigkeit vom Konservierungsmittel und dessen Einsatzkonzentration sowohl in vitro als auch in vivo antiseptische Effekte auftreten. So zeigt sich insbesondere für Propylenglykol, aber auch für das Parabengemisch, nicht nur in den In-vitro-Untersuchungen, sondern v. a. in der Proof-of-concept-Studie, eine Inhibition des Bakterienwachstums nach Konservierungsmittelapplikation. Auch wenn die vorliegenden Daten nicht durchgängig konstant sind, legen sie die Vermutung nahe, dass nicht nur bei einmaliger, sondern v. a. bei wiederholter Anwendung der Konservierungsmittel ein Einfluss auf die kutane Mikrobiota zu erwarten ist. Durch die begrenzte Fallzahl und die methodischen Schwierigkeiten ergeben sich zwar relevante Limitierungen der Aussagekraft, dennoch stützen die Proof-of-concept-Daten die Vermutung einer relevanten Interaktion zwischen Konservierungsmittel und kutaner Mikrobiota. Die wissenschaftliche Bedeutung dieser Assoziationsmuster ist bisher allerdings nur wenig beforscht. Deshalb bedarf es weiterer klinischer Studien, die sowohl größere Probanden- bzw. Patientenpopulationen als auch verschiedene Konservierungsmittel bzw. -gemische untersuchen und kulturelle, Sequenzierungs- und klinische Daten korrelieren.

Zusammenfassend zeigen die Ergebnisse der vorliegenden Proof-of-concept-Studie, dass von einer antiseptischen Wirkung von Konservierungsmitteln nach epikutaner Applikation auf der Hautoberfläche in Abhängigkeit vom verwendeten Substanzgemisch auszugehen ist. Sollte sich der Verdacht durch weiterführende Untersuchungen bestätigen, wäre es angebracht, den Einsatz von Konservierungsmitteln durch die gezielte Verwendung von galenischen Formulierungen mit kontinuierlicher lipophiler Außenphase zu beschränken, die Haltbarkeit von unkonservierten Topika zeitlich zu befristen bzw. den vermehrten Einsatz von rückschlagfreien Ventilen an Primärpackmitteln zu etablieren. Grundsätzlich ergeben sich aus dieser Thematik weitere Ansatzpunkte für eine neue Perspektive auf eine nachdrücklich zu fordernde, verbesserte Nachhaltigkeit topischer Formulierungen.
